# Recurrent Priapism Associated With Occupational Cocaine Exposure in a Patient With Sickle Cell Trait: A Case Report

**DOI:** 10.7759/cureus.101531

**Published:** 2026-01-14

**Authors:** Joel Powell, Hallie B Thompson, Steven J Laxton, Eric Bruno

**Affiliations:** 1 Emergency Medicine, University of Tennessee Health Science Center Nashville - Saint Thomas Health, Murfreesboro, USA

**Keywords:** acute ischemic priapism, cocaine-induced priapism, nonischemic priapism, priapism, substance-induced priapism

## Abstract

Cocaine is an uncommon but recognized cause of ischemic priapism due to its potent sympathomimetic and vasoactive properties. Case reports involving ischemic priapism related to intentional parenteral, intranasal, intracavernosal, and topical cocaine have been documented. Recurrent priapism resulting from occupational exposure to cocaine powder, however, has not been previously described. We report the case of a 37-year-old African American man with sickle cell trait who presented to the emergency department at least six times within a 12-month time period with recurrent priapism. Each episode was successfully detumesced with corporal aspiration and intracavernosal phenylephrine. The patient consistently denied recreational drug use; however, he admitted to working in the illicit drug industry “bagging” cocaine. This case highlights an unusual instance of recurrent ischemic priapism secondary to occupational cocaine exposure. Clinicians should maintain a high index of suspicion for environmental and occupational sources of cocaine absorption in patients presenting with unexplained or recurrent priapism. This case also highlights the first reported occupational exposure as a cause of priapism.

## Introduction

Priapism is defined as “a state of prolonged engorgement or erection of the penis or clitoris, not related to sexual desire or stimulation” [1]. Emergency department (ED) visits for priapism occurred at a rate of 5.34 per 100,000 male subjects per year, with the incidence being notably higher during summer months [2]. Although the incidence of spontaneous priapism remains low, high-risk populations include those with sickle cell disease, cocaine abuse, those who use intrapenile injections, pelvic malignancy, and those with perineal straddle injuries.

Priapism can be classified as ischemic (low-flow), nonischemic (high-flow), or stuttering (recurrent), referring to the arterial blood flow into the corpora cavernosa. High-flow priapism is characterized by increased arterial blood flow and is often associated with trauma. These are generally painless, nonemergent, and treated conservatively. Low-flow (ischemic) priapism, on the other hand, is a urologic emergency. The mechanism involves poor venous outflow, which causes increased intracavernosal pressure from trapped blood. This ultimately creates a type of compartment syndrome of diminished arterial blood flow, tissue ischemia/hypoxia, and penile pain [3]. Differentiating ischemic from nonischemic priapism generally begins with a detailed history and physical examination, as the presence of significant pain, certain underlying conditions (e.g., sickle cell disease), or certain medications can support the diagnosis of ischemic pathology. If the diagnosis is still unclear, performing penile blood gas analysis can aid in leading to a more definitive diagnosis.

Several etiologic factors have been associated with priapism, ranging from hypercoagulable/thromboembolic disorders (sickle cell disease, polycythemia, malignancy, etc.) to drugs, medications, and trauma. Data from the FDA pharmacovigilance database identified trazodone, olanzapine, and tadalafil as the three drugs most frequently linked to priapism [4]. Nonetheless, a wide range of substances, including antipsychotics, antidepressants, antihypertensives, marijuana, alcohol, and cocaine, have also been associated with ischemic priapism.

Cocaine use is a recognized, though uncommon, pharmacologic cause of priapism due to its vasoactive properties. Two primary mechanisms have been proposed to explain cocaine-induced priapism. The first involves cocaine’s inhibition of serotonin reuptake, resulting in vasodilation, a mechanism similar to that observed with selective serotonin reuptake inhibitors. The second proposed mechanism is the downregulation of adrenergic activity, specifically through reduced responsiveness of norepinephrine receptors, which also promotes vasodilation [5].

While cocaine-induced priapism following intentional use has been documented, recurrent priapism related to occupational exposure has not been previously reported. We present a rare case of recurrent ischemic priapism in a patient with sickle cell trait, secondary to chronic passive absorption of cocaine powder during occupational handling.

## Case presentation

A 37-year-old African American man presented to the ED for assessment of painful priapism that began about three hours before arrival. This was reportedly his fourth visit to the ED in the past 30 days for the same symptoms. He denied tobacco, alcohol, or illicit drug use and denied medication use or past medical history except for repeated priapism. Further questioning revealed that the patient had a history of sickle cell trait but no sickle cell disease. Physical examination revealed a rigid, erect penis with firm corpora cavernosa and a soft glans penis. Subjective penile pain without significant tenderness was noted. Laboratory findings in the ED are presented in Table 1.

**Table 1:** Emergency department laboratory results.

Test	Result	Reference range
Sodium (Na)	142 mmol/L	136–147 mmol/L
Potassium (K)	4.1 mmol/L	3.5–5.0 mmol/L
Chloride (Cl)	103 mmol/L	98–109 mmol/L
Bicarbonate (CO_2_)	31 mmol/L	22–32 mmol/L
Anion gap	8.0	6–16
Glucose	80 mg/dL	70–109 mg/dL
Blood urea nitrogen	3 mg/dL	9–22 mg/dL
Creatinine	0.9 mg/dL	0.8–1.4 mg/dL
White blood cell count	5.7 × 10^3^/mL	4.5–11.0 × 10^3^/mL
Red blood cell count	4.31 × 10^6^/µL	4.7–6.1 × 10^6^/µL
Hemoglobin	13.5 g/dL	13.5–17.5 g/dL
Hematocrit	39.8%	41–53%
Red cell distribution width	12.7%	11.5–14.5%
Platelet	306 × 10^3^/µL	150–450 × 10^3^/µL
Prothrombin time	12.5 seconds	11.2–13.9 seconds
International normalized ratio	1.0	0.9–1.1
Partial thromboplastin time	27.9 seconds	20–33 seconds
Urine amphetamines	Negative	Negative
Urine barbiturates	Negative	Negative
Urine benzodiazepines	Negative	Negative
Urine cannabinoids	Negative	Negative
Urine cocaine	Positive	Negative
Urine opiates	Negative	Negative
Urine phencyclidine	Negative	Negative
Urine tricyclics	Negative	Negative

When questioned about his urine drug screen positive for cocaine, the patient adamantly denied any recreational use of the drug. He did, however, admit to regularly handling cocaine, working as an illicit drug dealer “bagging” cocaine. The patient was successfully detumesced in the ED with subcutaneous terbutaline, needle aspiration, and manual compression, and he was discharged with instructions for cessation of handling of cocaine.

The patient returned to the same ED on five separate occasions following the initial visit, spanning over a course of about 12 months. In all but one of these visits, the priapism resolved following corporal aspiration and intracavernosal injection of phenylephrine, sometimes in combination with subcutaneous terbutaline. One episode of refractory priapism necessitated operative intervention with urology for further detumescence and continuous phenylephrine irrigation. He did have a repeat urine drug screen at this time, which was again positive for cocaine. The patient repeatedly denied any recreational or medicinal use of the substance, but disclosed his continued handling of cocaine with minimal protective gear.

Ultimately, the patient was discouraged from any further handling of cocaine. Following removal from this occupational environment, the patient did not experience further episodes of priapism during follow-up.

## Discussion

Cocaine-induced priapism is an uncommon clinical phenomenon resulting from the drug’s potent sympathomimetic and vasoactive effects. Priapism is classified into ischemic (low-flow) and nonischemic (high-flow) types, based on the hemodynamic characteristics of cavernosal blood flow. Ischemic priapism is a urologic emergency characterized by painful erections due to impaired venous outflow and subsequent tissue hypoxia, while high-flow priapism is painless and results from unregulated arterial inflow typically following trauma. In this case, the patient’s presentation, painful erections and response to aspiration and phenylephrine, is consistent with ischemic priapism.

The pathophysiology of cocaine-induced ischemic priapism is thought to involve disruption of sympathetic outflow and cavernosal smooth muscle tone. Cocaine works to inhibit the reuptake of norepinephrine, dopamine, and serotonin, causing sustained vasoconstriction, endothelial dysfunction, and impaired venous drainage [6,7]. Repeated or prolonged exposure to cocaine could paradoxically lead to cavernosal smooth muscle relaxation and venous trapping, creating conditions conducive to ischemia.

What distinguishes this case is that the patient repeatedly denied recreational use of cocaine, but admitted to daily handling and packaging of cocaine powder in an enclosed workspace. The consistent positive urine toxicology results raised the possibility of undisclosed personal use, initially a potential confounding factor; however, the patient’s consistent denial, absence of other substance use, and complete resolution of symptoms following cessation of occupational exposure strongly support passive occupational absorption as the primary cause. Exposure likely occurred through a combination of inhalation of airborne cocaine particulates and transdermal absorption through repetitive skin contact. Prior studies have demonstrated that individuals exposed to cocaine powder in occupational or environmental settings can develop measurable systemic absorption, even without direct use [8,9].

This case also raises the question regarding the minimum dose threshold and systemic absorption required to induce priapism. While this instance lacks the ability to quantify the minimum dose threshold, the patient’s case suggests that repeated low-dose exposure can produce sufficient systemic bioavailability to trigger the vasoactive effects of cocaine, even in the absence of direct use. This aids in broadening our understanding of cocaine’s toxicologic profile and suggests that priapism may occur at lower cumulative doses than previously recognized, particularly in individuals with predisposing conditions.

While sickle cell trait is less associated with recurrent priapism when compared to sickle cell disease, it may have acted as a predisposing cofactor, contributing to blood flow changes that increased susceptibility to ischemic events under vasoactive stress [10]. However, the association between the patient’s occupational exposure and the cessation of episodes following removal from that workplace environment supports cocaine as the primary etiologic factor.

This represents the first reported case of recurrent ischemic priapism secondary to occupational exposure to cocaine. It highlights the importance of healthcare providers keeping a broad differential diagnosis when seeing a patient with priapism and considering nontraditional routes of exposure, such as environmental and occupational sources, and to inquire specifically about contact with vasoactive substances, especially when toxicology results are positive, but the history does not align with recreational use.

## Conclusions

This case details a rare instance of recurrent ischemic (low-flow) priapism likely resulting from chronic occupational exposure to cocaine in a patient with sickle cell trait. While cocaine is a known cause of priapism, previous reports have almost exclusively originated from intentional recreational use. In contrast, this patient’s repeated episodes occurred in the setting of workplace exposure followed by complete resolution after discontinuing occupational contact with cocaine, exemplifying how passive, low-level occupational exposure may also result in systemic absorption significant enough to cause vascular complications such as priapism. It challenges clinicians to recognize that occupational or environmental contact with vasoactive substances can be a contributing factor in otherwise unexplained or recurrent priapism. Comprehensive social and occupational histories are essential in the evaluation of priapism, especially when routine causes are absent. Ultimately, early identification and elimination of exposure can prevent recurrence, preserve erectile function, and expand awareness of occupationally acquired vasoactive complications such as recurrent ischemic priapism.
